# GelMA Core–Shell Microgel Preparation Based on a Droplet Microfluidic Device for Three-Dimensional Tumor Ball Culture and Its Drug Testing

**DOI:** 10.3390/molecules30153305

**Published:** 2025-08-07

**Authors:** Xindong Yang, Yi Xu, Dongchen Zhu, Xianqiang Mi

**Affiliations:** 1 School of Microelectronics, Shanghai University, Shanghai 200444, China; yangxindong946@163.com; 2 Shanghai Institute of Microsystem and Information Technology, Chinese Academy of Sciences, Shanghai 200050, China; 3 School of Physics and Optoelectronic Engineering, Hangzhou Institute for Advanced Study, University of Chinese Academy of Sciences, Hangzhou 310024, China; 4 University of Chinese Academy of Sciences, Beijing 100049, China; 5 Human Phenome Institute, Fudan University, Shanghai 200438, China

**Keywords:** droplet microfluidic, GelMA, core–shell microgel, 3D cell culture, drug testing

## Abstract

Gelatin methacrylate (GelMA) microgels serve as promising bioscaffolds for tissue engineering and drug screening. However, conventional solid GelMA microgels often exhibit limited mass transfer efficiency and provide insufficient protection for embedded cells. In this study, we developed a droplet-based microfluidic platform to fabricate core–shell structured GelMA microgels. This system enabled precise control over microgel size and core-to-shell ratio by modulating flow rates. Encapsulation of A549 cells within these core–shell microgels preserved cellular viability and facilitated the formation of three-dimensional tumor spheroids. These outcomes confirmed both the protective function of the core–shell architecture during encapsulation and the overall biocompatibility of the microgels. The developed GelMA core–shell microgel system presents considerable applicability in research domains such as organoid modeling and high-throughput pharmacological screening.

## 1. Introduction

GelMA combines the advantages of both natural and synthetic materials. It incorporates natural arginine-glycine-aspartic acid sequences, which effectively mimic the extracellular matrix and support cellular adhesion [1,2]. In addition, its three-dimensional structure provides a suitable environment for cell proliferation and differentiation [3]. Owing to these characteristics, GelMA is widely regarded as an ideal biomaterial for the three-dimensional culture of various cell types [4,5]. It also offers excellent processing properties and forms crosslinked hydrogel networks through photopolymerization [6,7,8,9]. The formation and crosslinking of GelMA hydrogels can be temporally and spatially regulated by controlling ultraviolet (UV) light exposure [10].

Microgels are considered effective scaffolds in microtissue engineering due to their structural flexibility, softness, high colloidal stability, and extensive surface area, which enables multivalent biofunctionalization [11,12]. These features allow for the creation of microscale chambers, facilitating the assembly of diverse cell types into engineered microtissues [13].

Traditional methods for producing cell-laden microgels, including mechanical stirring [14], droplet suspension [14], and microarray printing [15], often result in oversimplified structures and poor structural stability. In contrast, droplet microfluidics allows precise manipulation of microgel dimensions, cellular encapsulation, and morphological control, making it a promising platform for tissue engineering and drug testing applications. Recent studies have explored the use of solid GelMA microgels in tissue construction [16,17,18,19,20,21], largely due to their favorable biocompatibility, biodegradability, cellular responsiveness, and three-dimensional architecture conducive to cell growth [22].

However, solid microgels often lack spatial complexity and tend to cause cellular damage during fabrication, which impedes effective three-dimensional cell aggregation. Moreover, numerous biological structures—such as blood vessels, the lens, kidneys, heart, lungs, and inner ear—develop through hollow morphogenesis [23,24,25,26,27,28]. Solid microgels do not accurately replicate these in vivo structural conformations [29]. In contrast, GelMA core–shell microgels possess a defined spatial organization and offer protective effects during formation, potentially addressing these limitations [30].

In this study, we developed a flow-focused droplet microfluidic chip for the fabrication of double-emulsion GelMA core–shell microgels. The chip established a stable double-emulsion laminar flow by controlling the gel phase (GelMA) and methyl cellulose (MC) phase through constant-flow syringe pumps. Fluorinated oil, introduced as the continuous phase, applied shear force to segment the flow and produced uniform double-emulsion droplets. These droplets were then subjected to emulsification to remove the oil shell, resulting in the formation of GelMA core–shell microgels. The resulting microgels were biocompatible and exhibited responsiveness to environmental conditions.

We first validated the system’s encapsulation capability using cultured cells. A549 cells were subsequently encapsulated in the microgels and cultured to form tumor spheroids. The platform was then employed to conduct drug response experiments using the generated spheroids.

## 2. Results and Discussion

### 2.1. Design and Fabrication of Droplet Microfluidic Chips

**Figure 1:**
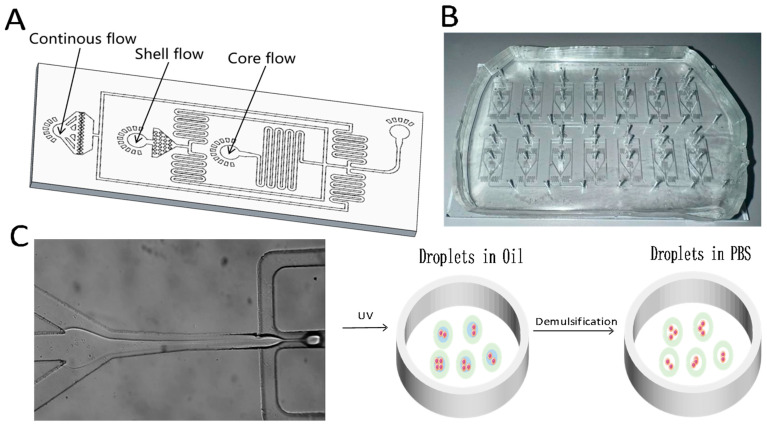
Schematic and physical layout of the droplet microfluidic chip used for generating core–shell microgels. (**A**) Chip layout overview. (**B**) Photograph of the fabricated device. (**C**) Diagram of the core–shell droplet generation process at the flow-focusing junction.

To produce core–shell microgels, we designed and fabricated a custom droplet microfluidic chip, as illustrated in Figure 1A,B. The device consists of two key components: a microgel generation unit and a collection port. The generation unit (Figure 1C) adopts a flow-focusing architecture with three inlets corresponding to the gel phase (GelMA), MC phase, and oil phase. This configuration allows for precise control over the laminar flow and facilitates the formation of double-emulsion droplets.

To maintain consistent inlet flow rates throughout the microgel generation process, we employed constant-flow syringe pumps. These pumps provide smooth, high-precision delivery, making them suitable for handling small-volume fluids. A serpentine channel was incorporated downstream to stabilize flow rates by balancing the fluidic resistance across all phases.

As shown in Figure 1, the system produces core–shell droplets within a water-in-oil-in-water (W/O/W) double-emulsion structure using the shear force generated by the oil phase. MC serves multiple functions in this setup. It is water-soluble, biocompatible, and physiologically inert, making it well-suited for cell culture applications [31,32,33]. In addition, it acts as a thickener, helping to suspend cells and stabilize the core stream during the formation of the hollow microgel structure [34]. The oil phase, composed of fluorinated oil, offers excellent biocompatibility and maintains high cell viability during the droplet formation process.

### 2.2. Optimization of Flow Rates in Droplet Microfluidic Chips

The flow rates of the three input phases in the droplet microfluidic system play a crucial role in determining the size and structural stability of the resulting microgels. To optimize these parameters, we systematically varied the flow rates of the GelMA, MC, and oil phases and recorded the corresponding diameters of both the nuclei and the full microgels. This evaluation also served to verify the flexibility and robustness of the chip.

**Figure 2:**
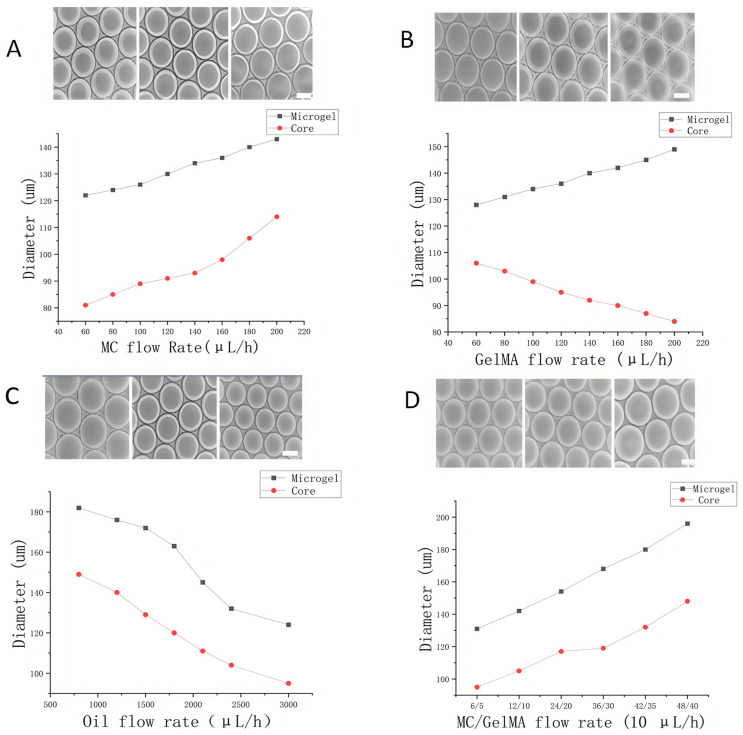
Influence of phase flow rates on microgel diameter and shell thickness. (**A**) Effect of MC phase flow rate. (**B**) Effect of GelMA phase flow rate. (**C**) Effect of oil phase flow rate. (**D**) Combined effect of MC and GelMA flow rates at a fixed oil phase flow rate (scale bar: 50 μm).

To assess the influence of the MC phase, its flow rate was incrementally increased from 60 μL/h to 200 μL/h, while the flow rates of the GelMA and oil phases remained constant. As shown in Figure 2A, both the nucleus and total microgel diameters expanded significantly with increasing MC flow rate. This is attributed to an elevated total flow rate of the continuous aqueous phases (MC and GelMA), which, relative to the oil phase, led to higher throughput through the flow-focusing junction. Consequently, a larger microgel volume formed, with both the nucleus and the outer diameter increasing in response to MC-guided flow expansion.

We then investigated the effect of the GelMA phase flow rate. While keeping the MC and oil phases constant, the GelMA flow rate was raised from 60 μL/h to 200 μL/h. As the GelMA flow increased, the nucleus diameter decreased from 112 ± 5.1 μm to 88 ± 4.6 μm, while the total microgel diameter increased from 127 ± 7.2 μm to 144 ± 3.6 μm (Figure 2B). This result supports the earlier conclusion: as the GelMA proportion increases, the continuous phase accelerates, thereby expanding the overall droplet diameter but reducing the core size under MC control. Similarly, modulation of the oil-phase flow rate revealed that both nucleus and microgel diameters decreased with increasing oil input (Figure 2C). This outcome was expected, as a higher shear rate from the oil phase would compress the dispersed phases more rapidly at the flow-focusing junction.

To further explore interactions between flow parameters, we simultaneously adjusted the flow rates of the MC and GelMA phases while keeping the oil-phase rate constant. This controlled experiment, conducted under a fixed flow rate ratio, demonstrated that increasing the combined flow rate of the aqueous phases led to enlarged core and shell dimensions (Figure 2D). These results affirm that microgel size and core–shell architecture can be finely tuned by manipulating the relative flow rates of all three input phases in the system.

## 3. Performance Validation of a Three-Dimensional Cell Culture System Based on a Droplet Microfluidic Chip

To construct a simulated three-dimensional cell culture microenvironment, the scaffold material was required to meet several essential criteria. First, it needed to allow effective permeability for nutrient and metabolic waste exchange to support physiological function and cellular metabolism [35]. Second, the material had to be biocompatible and should not trigger adverse effects such as toxicity, immune responses, or cellular stress upon contact [36]. The droplet microfluidic system used for cell encapsulation needed to maintain cellular viability during fabrication while preserving the functional characteristics of the scaffold material [37,38]. Therefore, we evaluated the permeability of GelMA core–shell microgels and the activity of encapsulated cells following the formation of cell-loaded droplets.

### 3.1. Permeability Testing of GelMA Core–Shell Microgels

Culturing cells within a core–shell microgel requires the scaffold to support efficient diffusion of nutrients and metabolic waste across the hydrogel interface. FITC-Dextran, a water-soluble fluorescent tracer widely used in biomedical applications, emits green fluorescence at specific wavelengths. It is frequently applied to evaluate the permeability of biological semi-permeable membranes [39,40,41]. To assess the permeability of the prepared GelMA core–shell microgels, we first calculated the diffusion coefficient (*D*) of FITC within our system, which was determined to be 1.2 × 10^−12^ m^2^/s based on previously published formulas [42,43]. This coefficient was calculated using the Stokes–Einstein equation:

(1) $$D=\frac{K_{B}T}{6\pi r\eta_{0}}$$

Here, *K_B_* represents the Boltzmann constant, η_0_ is the solvent viscosity, r is the diffusion radius, and *T* is the absolute temperature. In contrast, the diffusion coefficient of GelMA itself is typically approximated as 10^−11^ m^2^/s [44,45]. Following the diffusion coefficient calculation, we also developed a fluorescence-based imaging method to visualize and characterize the permeability of the GelMA core–shell microgels.

**Figure 3:**
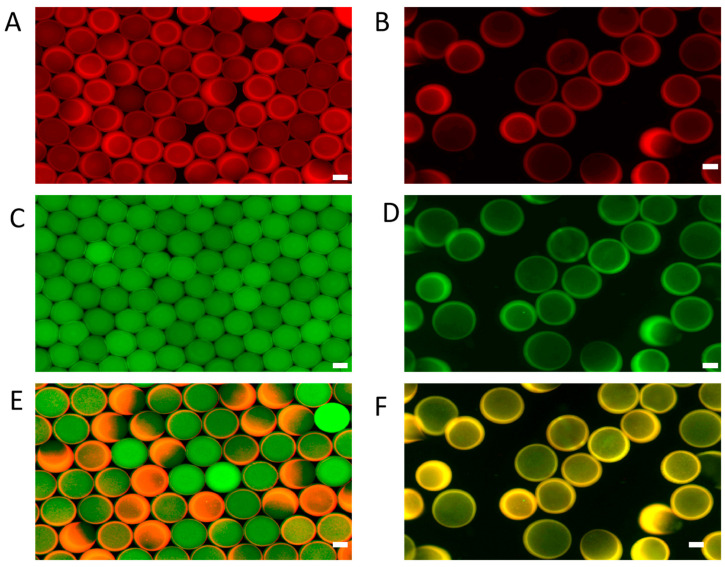
Characterization of GelMA core–shell microgel permeability. (**A**) GelMA shell before emulsion destabilization(red fluorescence); (**B**) GelMA shell after emulsion destabilization; (**C**) FITC-Dextran core before emulsion destabilization (green fluorescence); (**D**) Non-fluorescent core after emulsion destabilization; (**E**) Merged fluorescence image before emulsion destabilization; (**F**) Merged fluorescence image after emulsion destabilization (scale bar: 50 μm).

Double-emulsion droplets were generated using a solution containing high-molecular-weight FITC-Dextran and GelMA labeled with red fluorescence. These droplets were then photopolymerized to form solid gels before emulsion destabilization. During this process, the microgels suspended in fluorinated oil were transferred to a PBS aqueous solution. Representative fluorescence images of the GelMA shell, FITC-Dextran core, and their merged overlays are presented in Figure 3. Before emulsification, green fluorescent FITC-Dextran was localized within the core of the microgels, while red fluorescence from the GelMA matrix was uniformly distributed in the shell region. After emulsification, the green core fluorescence largely dissipated, whereas red fluorescence in the shell remained stable, indicating that FITC-Dextran diffused from the core into the surrounding aqueous phase. This observation confirms that the GelMA core–shell microgels are capable of exchanging solutes with their external environment, validating their potential use as three-dimensional cell culture vessels.

### 3.2. Morphological Characterization of GelMA Core–Shell Microgels

**Figure 4:**
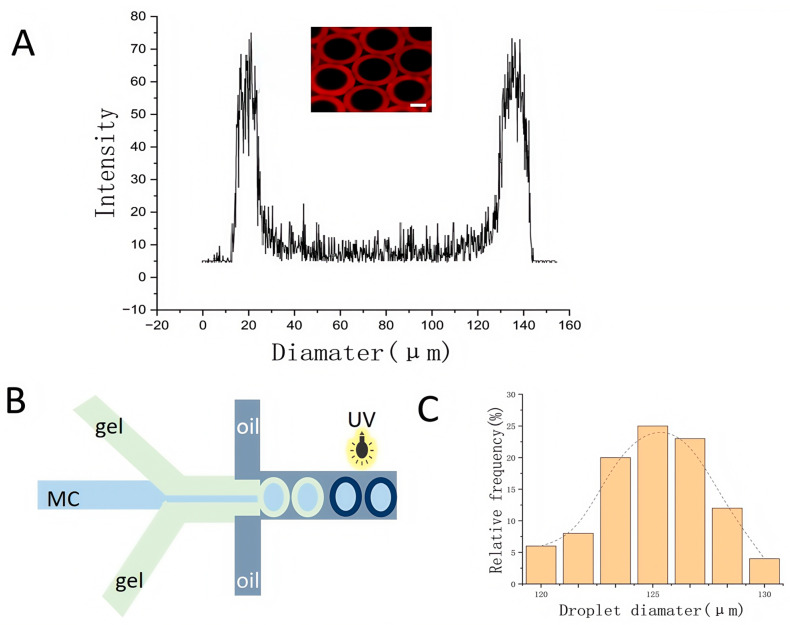
Morphological characterization of GelMA core−shell microgels. (**A**) Z−axis section and cross-sectional fluorescence intensity distribution of a core−shell microgel. (**B**) Schematic illustration of core−shell microgel formation. (**C**) Size distribution of the microgels (123 ± 4.2 μm). (Scale bar: 50 μm).

To verify the core–shell architecture of the GelMA microgels, GelMA was labeled with red fluorescent dye to visualize the core–shell structure. The structure of the core–shell microgel was analyzed using system-generated GelMA with red fluorescence, and the generation schematic is shown in Figure 4B. Confocal microscopy was used to analyze the structure of the core–shell microgel. Figure 4A shows a slice image of the core–shell microgel along the Z-axis and the corresponding fluorescence intensity distribution at the maximum cross-section, which confirms the presence of a clearly defined core–shell structure. In addition to this, in the present study, the size distribution of the empty microgels was also measured, as shown in Figure 4C.

### 3.3. Testing the Effect of Cell Encapsulation Process on Cell Activity Based on Droplet Microfluidic Chip

**Figure 5:**
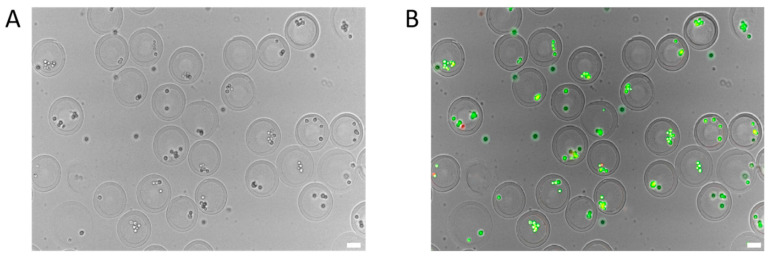
Droplet microfluidic chip for cell-loaded core–shell microgel generation. (**A**) Bright-field image of a cell-encapsulated microgel. (**B**) Fluorescence image of live/dead stained microgel; green indicates live cells, red indicates dead cells. (Scale bar: 50 μm).

The process of generating GelMA core–shell microgels and encapsulating cells using the designed droplet microfluidic chip must preserve cell viability throughout. To verify the cytocompatibility of our system, we conducted live/dead staining using calcein-AM and propidium iodide (PI) following the encapsulation procedure. As shown in Figure 5, the majority of encapsulated cells exhibited strong viability, indicated by green fluorescence, accounting for approximately 97% of the total cell population. A small number of red-stained cells, indicating cell death, were also observed, which likely originated from pre-existing non-viable cells in the input suspension. These findings confirm that the developed microfluidic system supports the generation of GelMA core–shell microgels while maintaining high cell viability, making it suitable for subsequent three-dimensional culture and biological applications.

## 4. Core–Shell Microgels as Carriers for Three-Dimensional Cell Culture

**Figure 6:**
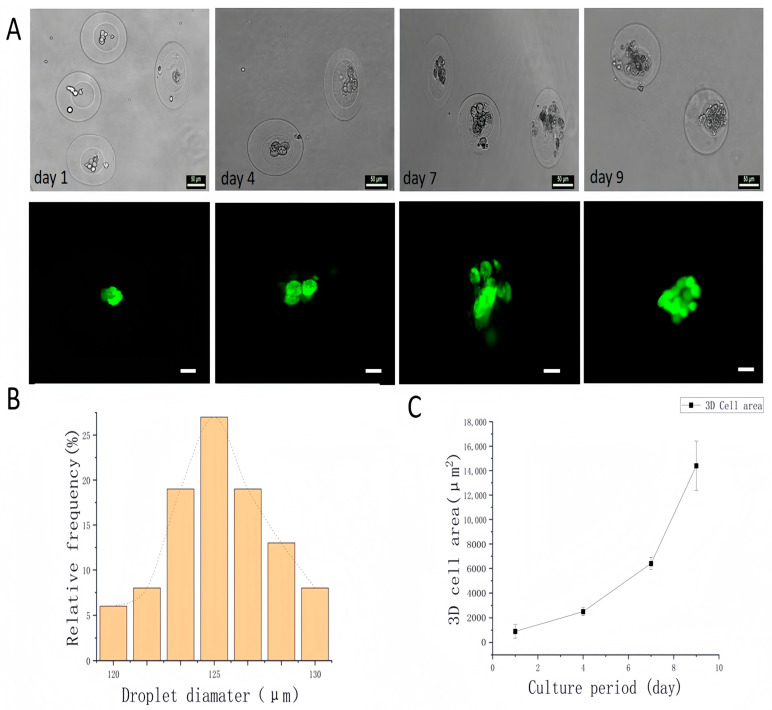
Evaluation of the viability and proliferation of A549 cells encapsulated in core–shell microgels. (**A**) Bright-field and fluorescence images of microgels on days 1, 4, 7, and 9; green fluorescence indicates live cells. (**B**) Size distribution of cell-loaded microgels (125 ± 5.2 µm). (**C**) Core diameter of microgels as a function of culture time. (Scale bar: 50 μm.).

To verify the biocompatibility of the prepared GelMA core–shell microgels for three-dimensional (3D) cell culture, A549 cells were loaded into the microgel cores after being mixed with the MC phase. The resulting cell-loaded droplets maintained a consistent size distribution and exhibited good monodispersity, reflecting the high generation stability of the system. As shown in Figure 6B, the average diameter of the cell-loaded microgels was 125 ± 9.1 µm, which was comparable to that of the empty microgels (123 ± 4.2 µm, Figure 4C).

Cell viability within the 3D microgel system was assessed using live–dead staining on days 1, 4, 7, and 9. As shown in Figure 6A, the encapsulated A549 cell spheroids maintained high viability throughout the culture period, confirming the biocompatibility of the platform. In addition, Figure 6C illustrates that the diameter of the 3D cell spheroids increased over time, indicating progressive cell proliferation. These results suggest that the droplet microfluidic system is capable of producing GelMA core–shell microgels with high uniformity and cytocompatibility, making them suitable for sustained 3D cell culture.

## 5. Drug Evaluation Based on Three-Dimensional Cells of Core–Shell Microgels

Compared with cells grown in two-dimensional (2D) cultures, three-dimensional tumor spheroids more closely replicate the architecture and physiological environment of actual tumor tissues. As such, they offered a more representative in vitro model for evaluating drug responses. To test this hypothesis, we evaluated the drug sensitivity of A549 tumor spheroids cultured in GelMA core–shell microgels using the chemotherapeutic agent doxorubicin (DOX), and compared the results with those from 2D-cultured A549 cells.

Doxorubicin is a clinically approved, broad-spectrum anticancer drug with a well-established mechanism of action, including DNA intercalation and inhibition of topoisomerase II [46,47,48]. It is widely recognized for its cytotoxic effects on cancer cells [49] and has been extensively tested in both 2D and 3D models, making it an appropriate choice for evaluating drug response in spheroid systems [50,51,52,53,54]. Furthermore, its intrinsic fluorescence and chemical characteristics enable future studies on drug penetration mechanisms within 3D cell structures [55].

**Figure 7:**
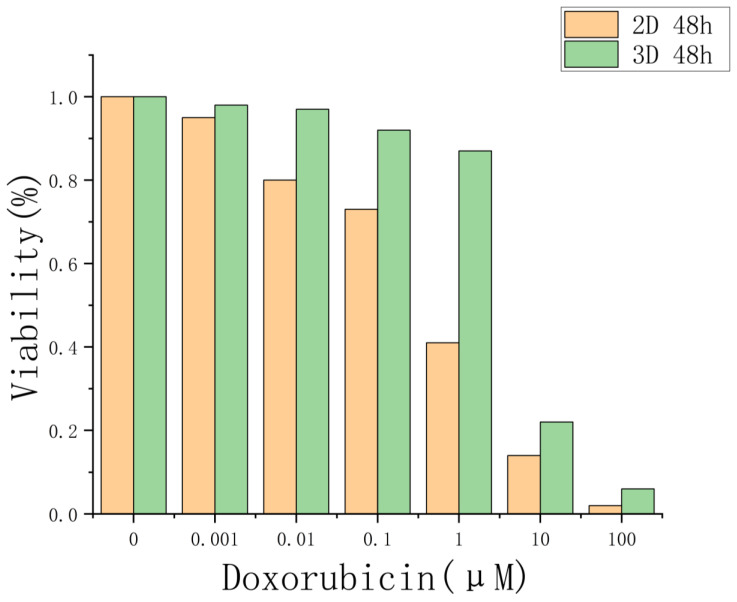
Comparison of drug sensitivity to doxorubicin (DOX) in 2D-cultured cells and 3D tumor spheroids derived from GelMA core–shell microgels.

As shown in Figure 7, we performed a CCK-8 cytotoxicity assay on 3D-cultured A549 cells maintained in 96-well plates for 9 days, as well as on 2D-cultured A549 cells treated with DOX for 48 h. The results demonstrated that increasing concentrations of DOX reduced cell viability in both culture systems. However, the 3D spheroids exhibited significantly greater resistance to the drug compared to their 2D counterparts, likely due to limited drug penetration into the spheroid core. These findings suggest that the drug response of 3D spheroids more accurately reflects in vivo drug sensitivity.

## 6. Experimental Section

Reagents: Gelatin methacryloyl (GelMA) and red fluorescent GelMA were obtained from Engineering for Life, Shanghai, China. The PEG–PFPE biocompatible surfactant was purchased from Zhejiang Dapu Biotechnology, Jiaxing, China. 1H-, 1H-, 2H-, and 2H-perfluorooctanol (PFO) was supplied by Macklin Biochemical Technology, Shanghai, China, while HFE7500 fluorinated oil was sourced from 3M Company, Saint Paul, MN, USA. FITC-Dextran (2000 kDa, diffusion radius ~1.2 nm) was provided by Maokang Biotechnology, Shanghai, China. Methyl cellulose (viscosity 1500 cP) was acquired from Sigma-Aldrich, Shanghai, China. All reagents were used without further purification.

Droplet microfluidic chip fabrication: Negative photoresist SU-8 was employed to fabricate the microfluidic molds. As a UV-curable material, SU-8 undergoes a cross-linking reaction when exposed to specific wavelengths of ultraviolet light, resulting in a solidified pattern. At the junction where the liquid phases converge, the channel widths were set at 80 µm for the inner-phase MC and the outer-phase hydrogel pre-solutions, 100 µm for the oil phase, and 150 µm for the remaining channel sections.

The microfluidic molds were created using two homogenization steps to achieve a pattern height of 130 µm. The first homogenization was conducted at 1200 rpm for 30 s. After a post-bake step, a second homogenization was performed at 1700 rpm for 30 s. Following homogenization, photolithography was carried out with a 30 s exposure to 365 nm UV light.

Polydimethylsiloxane (PDMS) monomer and curing agent were mixed at a mass ratio of 10:1 and poured into Petri dishes containing the microfluidic molds to form the channel structures. The PDMS layers were then plasma-bonded to produce sealed microfluidic chips. Finally, the surface of each chip was modified with a hydrophobic coating. The hydrophobic agent, primarily composed of 4-heptadecylidene-3-hexadecyloxetan-2-one, formed a nano-transparent layer on the channel surface, enhancing water contact angle and thereby facilitating droplet formation.

Generation of core–shell microgels: To fabricate GelMA core–shell microgels, the following solutions were injected into a droplet microfluidic chip using a constant-flow syringe pump (TJ-3A, Longer, Baoding, China). A 2% (*w*/*v*) PEG–PFPE solution in HFE7500 fluorinated oil was used for the oil phase; a 1.2% (*w*/*v*) methyl cellulose (MC) solution was used for the MC phase; and a 6% (*w*/*v*) GelMA solution was used for the Gel phase. For cell encapsulation, the flow rates were maintained at 120 μL/h for the MC phase, 150 μL/h for the Gel phase, and 1200 μL/h for the oil phase.

A polytetrafluoroethylene (PTFE) tube was used to connect the collection outlet of the chip to a centrifuge tube designated for capturing the generated microgels. The microgels in the collection tube were then photopolymerized by irradiation with 405 nm light at an intensity of 25 mW/cm^2^ (EFL-LS-1601-405, Engineering For Life) for 30 s to initiate GelMA crosslinking. The excitation wavelength of 405 nm was selected because it corresponds to the absorption characteristics of the GelMA photoinitiator, 2-Hydroxy-4′-(2-hydroxyethoxy)-2-methylpropiophenone, and causes minimal cellular damage [56].

After photocuring, an emulsion-breaking step was applied to release the microgels from the fluorinated oil phase. The emulsion breaker consisted of HFE7500 fluorinated oil mixed with 20% (*v*/*v*) 1H,1H,2H,2H-perfluorooctanol, which rapidly replaced the stabilizing film on the emulsion surface, thereby enabling the recovery of cured droplets for further analysis [57]. Following the addition of the emulsion breaker, the collected microgels were gently pipetted three times and centrifuged at 350 rpm for 1 min to remove any residual fluorinated oil and prepare the microgels for subsequent experiments involving substance exchange. All procedures and materials were handled under sterile conditions.

Permeability testing of GelMA core–shell microgels: FITC-Dextran is widely used to characterize the permeability of biological semi-permeable membranes [33,34,35]. To evaluate the permeability of GelMA core–shell microgels using FITC-Dextran (2000 kDa), the same microgel fabrication protocol was followed, with one modification: a red fluorescent GelMA pre-solution was used as the outer phase, while the inner phase solution was prepared by mixing 5% (*w*/*v*) FITC-Dextran (2000 kDa) with 1.2% (*w*/*v*) MC in PBS. The solution was vortexed for 1 min to ensure homogeneity. As the inner phase in the droplet formation process, the resulting microgel cores exhibited green fluorescence. Fluorescence images of the core–shell microgels were captured using a confocal microscope before and after the emulsion-breaking step to assess the diffusion of FITC-Dextran and the permeability characteristics of the GelMA shell.

Cell culture: The human non-small cell lung cancer cell line A549 was cultured in a humidified incubator at 37 °C with 5% CO_2_ and 90% relative humidity. Dulbecco’s Modified Eagle Medium (DMEM) supplemented with 10% fetal bovine serum (FBS) and 1% penicillin–streptomycin (PS) was used as the growth medium, and it was refreshed every two days.

When the cells reached approximately 90% confluence, passaging was performed. The old medium was removed using a pipette, and the adherent cells were rinsed one to two times with phosphate-buffered saline (PBS). A 0.25% EDTA–trypsin solution was added, and the dish was incubated at 37 °C for 2 min. The digestion was monitored under an inverted microscope. Once the cells had fully detached and formed a single-cell suspension, 1 mL of fresh medium was added to stop the enzymatic reaction. The entire suspension was transferred to a 1.5 mL centrifuge tube and centrifuged at 1000 rpm for 2 min. The supernatant was discarded, and the cells were resuspended in fresh medium. Gentle pipetting was applied until the cells were evenly dispersed. The resuspended cells were then seeded into new culture vessels at an appropriate dilution and returned to the incubator. Cell adherence was observed shortly after inoculation.

Cell encapsulation: To prepare cells for encapsulation, the culture medium was removed using a pipette, and adherent cells were washed one to two times with PBS. A 0.25% EDTA–trypsin solution was added and incubated at 37 °C for 2 min to dissociate the cells. The digestion process was terminated by adding 1 mL of fresh medium. The entire cell suspension was transferred to a 1.5 mL centrifuge tube and centrifuged at 1000 rpm for 2 min. After discarding the supernatant, the cells were resuspended in 1 mL of fresh medium and gently pipetted to ensure dispersion.

Next, 10 µL of the resuspended cell solution was loaded onto a Countess cell counting slide for cell enumeration using an automated cell counter. Based on the required concentration for the methyl cellulose (MC) mixture, the total volume of the resuspension was calculated. The sample was then centrifuged again at 1000 rpm for 2 min, the supernatant discarded, and 10 µL of fresh medium was added to minimize residual EDTA–trypsin activity. Finally, 1 mL of 1% MC solution was added, and the mixture was gently pipetted to ensure homogeneity. The final concentration of the cell–MC mixture used for encapsulation was adjusted to 1 × 10^7^ cells/mL.

Cell viability: The viability of A549 cells encapsulated within GelMA core–shell microgels was assessed after 1, 4, 7, and 9 days of culture using a live/dead staining kit (AM/PI). The microgel-encapsulated cells were washed three times with DPBS, stained with the AM/PI dye solution, and incubated at 37 °C for 20 min under dark conditions. Viable cells exhibited green fluorescence, while non-viable cells were stained red.

Drug sensitivity study of three-dimensional cultured tumor cell spheres: For the 2D drug testing procedure, A549 cells were seeded into 96-well plates at a density of 5 × 10^3^ cells per well, with approximately 100 μL of cell suspension per well. An automatic cell counter was used to standardize the inoculum, and six parallel experimental groups were established. After seeding, the cells were incubated for 48 h to ensure adherence. The test drug, doxorubicin (DOX), was diluted in DMEM according to a defined concentration gradient: 0.001 μM, 0.01 μM, 0.1 μM, 1.0 μM, 10.0 μM, and 100.0 μM. Each concentration was applied to the wells containing pre-cultured cells. A negative control group (no drug) was included, and each treatment had six replicates. The plates were incubated under constant temperature and humidity for 48 h to allow drug–cell interaction.

After the exposure period, the drug solutions were aspirated, and wells were rinsed once with 10 μL of PBS, which was then removed. Fresh medium (90 μL DMEM) and 10 μL of CCK-8 reagent were added to each well. The plates were returned to the incubator for 1 h, and absorbance was measured using a microplate reader to determine cell viability based on CCK-8 formazan production.

For the 3D drug testing protocol, A549-loaded GelMA core–shell microgels were cultured for 11 days. After incubation, the microgels were evenly divided into seven 2 mL centrifuge tubes and centrifuged to remove the medium. The same concentration gradient of DOX (0.001 μM to 100.0 μM) was prepared in culture medium, and 2 mL of each concentration was used to resuspend the corresponding microgel sample. Each suspension was transferred into a well of a 24-well plate and incubated for 48 h.

Following incubation, microgels were collected from each well, transferred to individual centrifuge tubes, and washed once with 1 mL PBS to remove residual drug. After a second centrifugation, the samples were resuspended in 1 mL of fresh medium. From each tube, six aliquots of 90 μL (each containing approximately 20 microgels) were dispensed into a 96-well plate. Subsequently, 10 μL of CCK-8 reagent was added to each well. The incubation and absorbance measurement procedures followed were identical to those used in the 2D assay.

## 7. Conclusions

This study presents a strategy for generating GelMA core–shell microgels using a droplet microfluidic chip. The size of the microgels could be readily adjusted by controlling the flow rates of the input phases. The resulting core–shell microgels exhibited favorable permeability characteristics. Moreover, the encapsulation process had no adverse effect on cell viability, allowing the embedded cells to be cultured over an extended period and to form stable three-dimensional structures.

The resulting 3D cultured cells maintained high viability and proliferative capacity. Drug sensitivity testing revealed that these 3D cell cultures displayed reduced responsiveness to chemotherapeutic agents compared to 2D monolayers, thereby offering a model that more closely reflects in vivo cellular responses. These findings demonstrated the protective and biocompatible nature of the GelMA microgel system and support its potential use in applications such as high-throughput organoid culture and downstream drug screening.

## Data Availability

The data presented in this study are available in the paper.
